# Transport and Recovery of Gilthead Seabream (*Sparus aurata* L.) Sedated With Clove Oil and MS-222: Effects on Stress Axis Regulation and Intermediary Metabolism

**DOI:** 10.3389/fphys.2019.00612

**Published:** 2019-05-31

**Authors:** Ismael Jerez-Cepa, Miriam Fernández-Castro, Thomas Julian Del Santo O'Neill, Juan Antonio Martos-Sitcha, Gonzalo Martínez-Rodríguez, Juan Miguel Mancera, Ignacio Ruiz-Jarabo

**Affiliations:** ^1^Department of Biology, Faculty of Marine and Environmental Sciences, Instituto Universitario de Investigación Marina (INMAR), Universidad de Cádiz, Campus de Excelencia Internacional del Mar (CEI·MAR), Puerto Real, Spain; ^2^Department of Marine Biology and Aquaculture, Institute of Marine Sciences of Andalusia (ICMAN), Spanish National Research Council (CSIC), Puerto Real, Spain

**Keywords:** welfare, *Sparus aurata*, anesthetics, transport, clove oil, MS-222, stress, intermediary metabolism

## Abstract

Transport processes between aquaculture facilities activate the stress response in fish. To deal with these situations, the hypothalamic-pituitary-interrenal (HPI) axis releases cortisol, leading to an increase in circulating energy resources to restore homeostasis. However, if the allostatic load generated exceeds fish tolerance limits, stress-related responses will compromise health and welfare of the animals. In this context, anesthetics have arisen as potential agents aiming to reduce negative effects of stress response. Here we assessed the effects of a sedative dose of clove oil (CO) and MS-222 on hallmarks involved in HPI axis regulation and energy management after simulated transport, and further recovery, in gilthead seabream (*Sparus aurata* L.) juveniles. Fish were placed in a mobile setup of water tanks where transport conditions were simulated for 6 h. Sedation doses of either CO (2.5 mg L^−1^) or MS-222 (5 mg L^−1^) were added in the water tanks. A control group without anesthetics was also included in the setup. Half of the animals (*n* = 12 per group) were sampled immediately after transport, while remaining animals were allowed to recover for 18 h in clean water tanks and then sampled. Our results showed that the HPI axis response was modified at peripheral level, with differences depending on the anesthetic employed. Head kidney gene-expressions related to cortisol production (*star* and *cyp11b1*) matched concomitantly with increased plasma cortisol levels immediately after transport in CO-sedated fish, but these levels remained constant in MS-222-sedated fish. Differential changes in the energy management of carbohydrates, lipids and amino acids, depending on the anesthetic employed, were also observed. The use of CO stimulated amino acids catabolism, while MS-222-sedated fish tended to consume liver glycogen and mobilize triglycerides. Further studies, including alternative doses of both anestethics, as well as the assessment of time-course HPI activation and longer recovery periods, are necessary to better understand if the use of clove oil and MS-222 is beneficial for *S. aurata* under these circumstances.

## Introduction

Welfare of farmed fish is of concern for aquaculture industry due to its effects on production efficiency and related economic benefits (Sneddon et al., 2016). The routine husbandry and management processes in aquaculture facilities, e.g., handling, stocking density or transport, can result in an activation of the stress system and thus compromise animal welfare (Ashley, 2007; Sneddon et al., 2016). Primary stress responses in teleost fish are mediated by the activation of the hypothalamic-sympathetic-chromaffin (HSC) axis and the hypothalamic-pituitary-interrenal (HPI) axis, with the consequent release of catecholamines and cortisol into the blood, respectively (Wendelaar Bonga, 2011; Schreck and Tort, 2016). HSC axis is actually considered the proper stress axis, whereas HPI axis regulation and all the physiological responses derived from cortisol actions aimed at the acclimation of the fish (Koolhaas et al., 2011). Cortisol release starts by up-regulation of the neurohypothalamic factor corticotrophin-releasing hormone (CRH), whose levels are regulated by the CRH-binding protein (CRHBP) (Flik et al., 2006), and also by the thyrotropin-releasing hormone (TRH) (Ruiz-Jarabo et al., 2018). These factors control the release of proopiomelanocortin-derived hormones (POMCs) at hypophyseal level, like the adrenocorticotropic hormone (ACTH), into the bloodstream (Flik et al., 2006). Finally, ACTH stimulates cortisol synthesis in the interrenal cells of the head kidney through the activation of key enzymes such as the steroidogenic acute regulatory protein (StAR) and the 11β-hydroxylase (Cyp11b1) (Montero et al., 2015; Skrzynska et al., 2018).

In teleost fish, cortisol has a dual adaptive role as glucocorticoid and mineralocorticoid hormone, mediated by its interaction with gluco- and mineralo-corticoid receptors (Faught et al., 2016). However, their interactions and the physiological responses derived from different stress situations are still largely unknown (Kiilerich et al., 2018; Tsalafouta et al., 2018). Classically, the mineralocorticoid action of cortisol improves osmoregulatory performance of gills, the main tissue involved in ion-balance in fish, by regulating the activity of several ion pumps such as the Na^+^/K^+^-ATPase (NKA) (Mancera et al., 2002; Takei and McCormick, 2013) and the permeability of the epithelia (Kelly and Chasiotis, 2011). As a glucocorticoid, cortisol stimulates the intermediary metabolism and mobilizes energy to deal with the stress situation (Mommsen et al., 1999; Vijayan et al., 2010). Cortisol induces plasma hyperglycemia through breakdown of stored hepatic glycogen and increases gluconeogenesis from alternative non-glycolic sources (Barton, 2002), as triglycerides (TAG), free fatty acids (FFA), and amino acids to provide energetic substrates (Faught and Vijayan, 2016). Thus, the metabolic effects of cortisol are key players involved in fish welfare.

In this context, different anesthetics have arisen as a solution to improve animals' welfare during aquaculture procedures. Some of them, including benzocaine, MS-222 (tricaine methanesulfonate), clove oil, metomidate, isoeugenol, 2-phenoxyethanol, quinaldine, or ketamine are widely employed for different fish species (Ross and Ross, 2008; Priborsky and Velisek, 2018). These anesthetics have been proved useful for practical procedures in aquaculture processes such as class-size sorting, weighting, sampling, surgeries, or breeders manipulation (Weber et al., 2009; Toni et al., 2015). Their employment at sedation doses was also proposed during transport to minimize stress and improve welfare (Ashley, 2007; Sneddon et al., 2016). Nevertheless, fish responses to anesthetics are species-specific and dependant on compounds' chemical properties (Readman et al., 2017).

In Europe, the use of anesthetics in fish for human consumption is restrictive, and only MS-222, benzocaine and isoeugenol are approved (European Commission, 2010, 2011), while in the USA only MS-222 (ANADA 200-226) is permitted (FDA, 1997). However, the use of anesthetics during fish transport is still limited (European Council, 2005). In these terms, transport standards of the World Organization for Animal Health are focused on management and water quality, while sedation is not considered necessary to guarantee fish welfare (OIE, 2018). Despite this, a slight sedation during transport has proved to reduce the metabolic rate of fish, and consequently oxygen consumption and generation of waste products, improving water quality management (Zahl et al., 2012; Vanderzwalmen et al., 2018). Although some of these compounds can generate additional effects on the stress response of fish, such as increased cortisol levels, altered oxidative stress status and immune system, or even decreased food intake (Ortuño et al., 2002; Pirhonen and Schreck, 2003; Azambuja et al., 2011), further studies are necessary to evaluate their putative benefits during fish transport processes, as well as after a recovery period.

The purpose of this study was to determine the physiological effects of a sedation dose of MS-222 or clove oil in transported gilthead seabream (*Sparus aurata*) juveniles. Changes in mRNA levels of the main HPI factors in brain, pituitary and head kidney, and plasma cortisol were analyzed. Furthermore, intermediary metabolism in liver was assessed to determine changes in the management of carbohydrates, lipids and amino acids due to anesthetics' addition. MS-222 and clove oil are probably the most widely used anesthetics in the world, but their physiological effects may show differences depending on many factors (reviewed in Priborsky and Velisek, 2018). The results from this study will contribute to elucidate if these compounds improve fish welfare in transport processes related to aquaculture of *S. aurata*.

## Materials and Methods

### Animal Maintenance

Fish were provided by *Servicios Centrales de Investigación en Cultivos Marinos* (SCI-CM, CASEM, University of Cadiz, Puerto Real, Cádiz, Spain; Spanish Operational Code REGA ES11028000312). Immature gilthead seabream (*S. aurata*) juveniles (*n* = 160, 42.7 ± 6.8 g body mass, mean ± SD) were transferred to the facilities of the Department of Biology at the Faculty of Marine and Environmental Sciences (CASEM, University of Cadiz, Puerto Real, Cádiz, Spain) and acclimated to laboratory conditions for 7 days in four flow-through 500 L tanks (approximate stocking density: 3.5 kg m^−3^), with seawater in controlled conditions of salinity (38 ppt) and temperature (19 °C), and under natural photoperiod (May 2015; 13:11 h, light:dark; 36°31′45″ N, 6°11′31″ W). Animals were fed twice daily (1 % of tank biomass per day) with commercial pellets for *S. aurata* (Skretting España S.A., Spain). Fish were kept and handled following the guidelines for experimental procedures in animal research from the Ethics and Animal Welfare Committee of the University of Cadiz, according to the Spanish (RD53/2013) and European Union (2010/63/UE) legislation. The Ethical Committee from the Autonomous Andalusian Government approved the experiments (Junta de Andalucía reference number 28-04-15-241).

### Characterization of Exposure to Anesthetics

*S. aurata* juveniles were exposed to different concentrations of clove oil (CO, extracted from cloves of *Eugenia* spp., Sigma-Aldrich C8392) and buffered MS-222 (tricaine methanesulfonate, Sigma-Aldrich E10521) to determine the optimal sedation dose for the simulated transport assay. Fish were transferred individually into 5 L glass-aquaria within different concentrations of CO: 5, 10, 20, 40, 60 mg L^−1^; or MS-222: 10, 25, 50, 70, 80, 100 mg L^−1^ (*n* = 8). Doses selected were in accordance to other authors for the same species, fish size and water temperature (Mylonas et al., 2005; Vera et al., 2010). Biochemical conditions of the water employed were the same as those in the acclimation tanks. All aquaria were oxygen-saturated (> 90% O_2_ saturation) with fine bubbles from an air stone to ensure maximum gas exchange efficiency. To avoid increased metabolic rates and O_2_ consumption, animals were fasted for 24 h before anesthetics exposure. Water and anesthetic were freshly renewed for each fish to avoid metabolic waste accumulation in the aquaria and ensure concentration, respectively. Once each fish was placed into the aquarium, induction times to sedation and anesthesia were recorded for each stage. Two different grades, light and deep, were defined also for both sedation and anesthesia stages. The exposure was finished when fish either reached all the stages or after 30 min without changes in the induction progress. Then, fish were transferred to a clean-water aquarium to determine the recovery time. The progressive stages of the induction and recovery, defined in Supplementary File 1, were adapted from Ross and Ross (2008).

### Simulated Transport and Sampling Procedure

Fish were randomly placed into a mobile setup of nine 15 L aquaria, and distributed in three different experimental groups in triplicate (*n* = 72). Then, *S. aurata* juveniles were transported for 6 h with either 2.5 mg of CO L^−1^ or 5 mg of MS-222 L^−1^, plus a control group without anesthetics. The selected doses of anesthetics were half of the lowest concentration that induced a light sedation in the previous described characterization protocol (section Characterization of Exposure to Anesthetics in this manuscript; detailed results and explanations are provided in the results section). All aquaria were oxygen-saturated (> 90% O_2_ saturation) with air stones. Animals were fasted for 24 h before the assay. To simulate transport conditions, every 20 min the mobile setup of aquaria was displaced for 5 min (mimicking noise and vibrational disturbances due to shaking) followed by 15 min of resting. After 6 h half of the animals were euthanized and sampled (*n* = 12 per experimental group), whereas the remaining fish were transferred to similar clean-water aquaria, allowed to recover for 18 h, and then euthanized and sampled as well. No food was supplied during recovery period (in total, animals were fasted for 48 h maximum). Sampling times were selected according to virtual industrial conditions. Thus, the transport started at 9 a.m. and finished at 5 p.m., when animals were sampled. The final end point (18 h after start of the recovery period) was selected as in this species, complete physiological recovery after an acute stress challenge occurs 6 h later (Skrzynska et al., 2018). Thus, the last sampling point was conducted at the same time of the day as the transport started, aiming at the emulation of a regular workday in aquaculture facilities.

Sampled animals were netted and deeply anesthetized with 2-phenoxyethanol (1 mL L^−1^, Sigma-Aldrich 77699), to standardize the stunning protocol in all experimental groups. 2-phenoxyethanol was selected due to its low time to induce deep anesthesia (< 1 min) without significant effects on the physiological parameters assessed (Toni et al., 2015; Priborsky and Velisek, 2018). Then fork length and body mass were recorded, and blood collected from caudal vessels with ammonium-heparinized syringes (Sigma-Aldrich H6279, 25000 units in 3 mL of saline 0.9 % NaCl). Plasma was separated from cells by centrifugation of blood (3 min, 10000 × *g*, 4°C) and snap frozen in liquid nitrogen. Fish were subsequently euthanized by spinal cord sectioning. Whole brain, pituitary and representative portions from head kidney were collected, placed into tubes with 10-volumes (v/w) of RNA*later*™ (Invitrogen by Thermo Fisher Scientific), held for 24 h at 4°C and then stored at −20°C until total RNA isolation. Liver was also excised, and the portions collected in microtubes were snap frozen in liquid nitrogen and stored at −80°C until metabolites quantification and enzymatic activities assessment. Additionally, the second gill arch on the left side was also excised, adherent blood was removed by blotting with absorbent paper and few branchial filaments collected and placed into microtubes with 100 μL of ice-cold sucrose-EDTA-imidazole (SEI) buffer (150 mM sucrose, 10 mM EDTA, 50 mM imidazole, pH 7.3) for analysis of Na^+^/K^+^-ATPase (NKA) activity.

### Total RNA Isolation and mRNA Levels

Total RNA from brain and head kidney portions were isolated with NucleoSpin® RNA kits (Macherey-Nagel), whereas pituitaries were processed with NucleoSpin® RNA XS kits (Macherey-Nagel). To reduce gDNA contamination, an on-column rDNase digestion was carried out according to kits' specifications (Macherey-Nagel) on each sample. RNA quality was determined using a 2100 Bioanalyzer (Agilent Technologies), and total RNA quantified in a Qubit® 2.0 Fluorometer with Qubit™ RNA BR Assay Kit (Invitrogen by Thermo Fisher Scientific). Only samples with best RNA Integrity Number (RIN > 8.0) were used for real-time PCR (qPCR). Before mRNA expression levels determination, RNA was reverse transcribed using a qSCRIPT™ cDNA Synthesis Kit (Quanta BioSciences™). Samples from brain and head kidney were equaled to 500 ng of RNA in a final volume of 20 μL for the cDNA synthesis, whereas for pituitary 50 ng of RNA were used.

The qPCR was performed by semi-quantitative fluorescence with a CFX Connect™ Real-Time PCR System (Bio-Rad Laboratories) in 96 white wells Hard-Shell® PCR plates covered with Microseal® “B” Seals (Bio-Rad). On each well, the total reaction mixture of 10 μL contained 0.5 μL of each specific reverse and forward primers, 5 μL of PerfeCTa SYBR® Green FastMix™ 2x (Quanta BioSciences™), and 4 μL of cDNA from each sample. The amount of cDNA template was 1 ng for pituitaries, and 10 ng for brain and head kidney samples. Primers for *crh* (GenBank acc. no. KC195964), *crhbp* (acc. no. KC195965), *trh* (acc. no. KC196277), *pomc*α*1* (acc. no. HM584909), *pomc*α*2* (acc. no. HM584910), and *star* (acc. no. EF640987) were used as described other works, from our research group, for *S. aurata* (Martos-Sitcha et al., 2014; Toni et al., 2015; Ruiz-Jarabo et al., 2018; Skrzynska et al., 2018). For *11*β*-hydroxylase* (*cyp11b1*, acc. no. FP332145), and *glucocorticoid receptor* (*nr3c1*, acc. no. DQ486890) genes, primers annealing temperature (50–60 °C), primers concentration (100, 200 and 400 nM), and template concentration (1:10 serial dilutions of cDNA, from 10 ng to 100 fg) were tested to optimize qPCR conditions. Two negative controls: NRT (no reverse transcriptase, 10 ng RNA/reaction), and NTC (no template control, only Tris-HCl 10 mM [pH 8.0], 0.1 mM EDTA) were also added to detect, respectively, possible gDNA contamination or primer-dimer by-products of PCR. Curves with 1:10 serial dilutions of template concentration (from 10 ng of cDNA to 100 fg for brain and head kidney; and from 1 ng to 10 fg for pituitary samples) were performed to test linearity and efficiency of each pair of primers. The reaction protocol for qPCR was conducted in the detection system as follows: 95°C, 10 min; [95°C, 15 s; 60°C, 30 s] × 40 cycles; plus melting curve ([from 60 to 95°C, 0.5°C per read, 70 reads], 95°C, 15 s) to ensure the amplification of a single product and the non-appearance of primer-dimers. Results were normalized to two reference genes, β*-actin* (*actb*, acc. no. X89920) and *elongation factor 1*α (*ef1a*, acc. no. AF184170), due to their low variability in our experimental conditions (M-value < 0.5). Stability M-values of reference genes were 0.1491, 0.1490, and 0.2391 for brain, pituitary and head kidney, respectively. Relative gene expression was performed by ΔΔCq Normalize Expression Gene Study with Bio-Rad CFX Manager™ 3.1 software. Nucleotide primers designs and amplicon sizes, as well as efficiencies and R^2^ from serial dilution curves are summarized in Supplementary File 2.

### Plasma Parameters

Plasma cortisol levels were measured with a commercial Cortisol Enzyme Immunoassay Kit from Arbor Assays™ (NCal™ International Standard Kit, DetectX®, K003). Glucose, lactate and triglyceride levels in plasma were measured using commercial kits from Spinreact (St. Esteve de Bas, Girona, Spain) adapted to 96-well microplates. Plasma total protein concentration was determined with a BCA Protein Assay Kit (Pierce™, Thermo Fisher Scientific, USA, #23225) using BSA as a standard. Total α-amino acid levels were assessed colorimetrically using the ninhydrin method from Moore (1968) adapted to 96-well microplates. Plasma ammonium was measured following the method from Bower and Holm-Hansen (1980) adapted to 96-well microplates, wherein ${\text{NH}}_{4}^{+}$ reacts with salicylate and hypochlorite to form a spectrophotometrically measurable adduct at 650 nm. The method was validated through serial dilutions of *S. aurata* plasma samples with 0.6 % (w/v) NaCl solution at pH 7.2, to ensure plasma ammonia balance. The method resulted in a confident linearity up to 60 μM for 1:500 diluted plasma of *S. aurata*. All assays were performed using a PowerWave™ 340 microplate spectrophotometer (Bio-Tek Instruments, Winooski, VT, USA) using KCjunior™ data analysis software for Microsoft®.

### Liver Parameters

Frozen liver samples were finely minced on an ice-cooled Petri dish and divided into two aliquots to assess enzyme activities and metabolite levels. The frozen tissue used for the assay of metabolites was homogenized by ultrasonic disruption in 7.5 volumes ice-cold 0.6 N perchloric acid, neutralized using 1 M KCO_3_, centrifuged (30 min, 3220 × *g* and 4°C), and then the supernatant isolated to determine tissue metabolites. Tissue triglycerides levels were determined spectrophotometrically with commercial kits (Spinreact, see before). Tissue glycogen concentration was quantified using the method from Keppler and Decker (1974). Glucose obtained after glycogen breakdown with amyloglucosidase (Sigma-Aldrich A7420) was determined with a commercial kit (Spinreact, see before). Total α-amino acid levels were assessed colorimetrically with the ninhydrin method as described above for plasma samples.

Frozen liver portions for enzymatic activities assays were homogenized by ultrasonic disruption in 10 volumes of ice-cold homogenization buffer (50 mM imidazole, 1 mM 2-mercaptoethanol, 50 mM NaF, 4 mM EDTA, 0.5 mM phenylmethylsulfonyl fluoride (PMSF) and 250 mM sucrose; pH 7.5). The homogenate was centrifuged for 30 min at 3220 × *g* and 4°C, and the supernatant stored at −80°C for further analysis. The assays of GP (glycogen phosphorylase, EC 2.4.1.1), PK (pyruvate kinase, EC 2.7.1.40), GLDH (glutamate dehydrogenase, EC 1.4.1.2), AST (aspartate aminotransferase, EC 2.6.1.1), ALT (alanine aminotransferase, EC 2.6.1.2), G6PDH (glucose-6-phosphate dehydrogenase, EC. 1.1.1.49), HADH (3-hydroxyacyl-CoA dehydrogenase, EC 1.1.1.35), GPDH (glycerol-3-phosphate dehydrogenase, EC 1.1.1.8), FBP (fructose 1,6-bisphosphatase, EC 3.1.3.11), and LDH-o (lactate dehydrogenase-oxidase, EC 1.1.1.27), were performed as previously described for *S. aurata* (Laiz-Carrión et al., 2003; Sangiao-Alvarellos et al., 2005, 2006; Polakof et al., 2006; Vargas-Chacoff et al., 2016). Enzyme activities were determined using a PowerWave™ 340 microplate spectrophotometer (Bio-Tek Instruments, Winooski, VT, USA) using KCjunior™ data analysis software for Microsoft®. Reaction rates of enzymes were determined by changes in absorbance from the reduction of NAD(P)^+^ to NAD(P)H, measured at 340 nm and 37 °C, during pre-established times (10-15 min). Activities were referenced to protein content of homogenate (U mg prot^−1^). Proteins were assayed in duplicate, as described for plasma samples.

### Osmoregulation Parameters

Plasma osmolality was measured with a vapor pressure osmometer (Fiske One-Ten, Fiske, VT, USA) and expressed as mOsm kg^−1^. Na^+^/K^+^-ATPase (NKA) activity in gills was determined in 96-well microplates as Mancera et al. (2002) performed for *S. aurata* based on the method described my McCormick (1993). The reactions were allowed to proceed at 25°C and changes in absorbance were monitored during pre-established times (5–10 min). Proteins were assayed in duplicate, as described for plasma samples.

### Statistics

One-way ANOVA was performed for each anesthetic and stage during the induction progress (8 fish per treatment). The other variables were evaluated in 12 animals per experimental group and time (four animals per aquarium, in triplicate). Two-way nested ANOVA was performed to evaluate inter-tank variability of replicates for all parameters at each sampling point. Since no significant variability was determined due to triplicates in any of the dependent variables (*p* ≥ 0.25), tanks were subsequently treated as a single group. When necessary, data were logarithmically transformed to fulfill the requirements for parametric statistical analysis. Gaussian distribution was confirmed using the Kolmogorov-Smirnov's test. The homogeneity of variances was analyzed by Levene's test. The effect of the anesthetics doses (none, clove oil or MS-222), and sampling times (after stress or after recovery), as well as its possible interaction in parameters assessed was tested using two-way ANOVA, with “anesthetic” and “time” as main factors. Tukey's *post hoc* test was used to identify significantly different groups. A Linear Regression Model was performed to determine correlation between *cyp11b1* and *star* mRNA levels. Statistical significance was accepted at *p* < 0.05. All the results are given as mean ± standard error of the mean (SEM).

## Results

### Characterization of Exposure to Anesthetics

Induction times to sedation and anesthesia stages in *S. aurata* juveniles showed a dose-dependent variation for both clove oil (CO) and MS-222 (Table 1). Animals exposed to CO reached deep anesthesia above 20 mg L^−1^, requiring almost 9 min at this concentration. However, this stage is reached in < 3 min at 40 mg L^−1^ (159 ± 18 s), while taking significantly less time at 60 mg L^−1^ (80 ± 5 s, *p* = 0.0001). Recovery times at both highest concentrations of CO were similar, 359 ± 57 s and 403 ± 26 s respectively (*p* = 0.9046). MS-222 induced deep anesthesia above 70 mg L^−1^, requiring more than 9 min at this concentration, and < 3 min at 80 mg L^−1^ (214 ± 9 s) and 100 mg L^−1^ (202 ± 15 s), without significant differences between the two highest concentrations (*p* = 0.8032). However, recovery time at 100 mg L^−1^ (525 ± 22 s) was significantly higher (*p* = 0.0186) than at 80 mg L^−1^ (290 ± 19 s). The lowest concentration of CO (5 mg L^−1^) only induced light sedation after 12 min of exposure, and that of MS-222 (10 mg L^−1^) only induced light sedation after 14 min of exposure. Consequently, half of those concentrations were selected for the simulated transport, 2.5 mg L^−1^ for CO and 5 mg L^−1^ for MS-222, to ensure that animals did not get a deeper sedation stage during the whole 6 h transport process. No mortality occurred due to anesthetics exposure.

**Table 1:** Characterization of the induction progress in *S. aurata* juveniles exposed to different concentrations of clove oil and MS-222.

**Anesthetic (mg L^**−1**^)**	**Sedation stage**		**Anesthesia stage**		**Recovery**
	**Light**	**Deep**	**Light**	**Deep**	
**CLOVE OIL**					
5	730 ± 159A	*n*.*r*.	*n*.*r*.	*n*.*r*.	325 ± 31*B*
10	79 ± 10B	146 ± 15A	*n*.*r*.	*n*.*r*.	1040 ± 26A
20	47 ± 3BC	83 ± 14B	109 ± 6A	528 ± 44A	427 ± 30B
40	34 ± 5C	55 ± 9BC	60 ± 8B	159 ± 18B	359 ± 57B
60	17 ± 1D	27 ± 2C	36 ± 2C	80 ± 5C	404 ± 26B
**MS-222**					
10	854 ± 126A	*n*.*r*.	*n*.*r*.	*n*.*r*.	173 ± 28D
25	281 ± 11B	*n*.*r*.	*n*.*r*.	*n*.*r*.	354 ± 67BC
50	110 ± 5C	234 ± 6A	1014 ± 109A	*n*.*r*.	455 ± 52AB
70	72 ± 2D	112 ± 4B	268 ± 19B	547 ± 56A	273 ± 20CD
80	40 ± 4E	57 ± 4C	116 ± 15C	214 ± 9B	289 ± 19CD
100	28 ± 4F	44 ± 4C	75 ± 17D	202 ± 15B	525 ± 22A

*Times (s) of induction to light and deep sedation and anesthesia, as well as recovery are represented as mean ± SEM (n = 8). n.r.: not reached stage. Different sets of capital letters represent statistical differences among doses for each stage of the induction progress (one-way ANOVA followed by a post-hoc Tukey test, p < 0.05)*.

### Simulated Transport With Sedation Doses of Anesthetics

*S. aurata* juveniles transported under sedative conditions, with CO (2.5 mg L^−1^) or MS-222 (5 mg L^−1^), presented modifications in the HPI axis regulation as well as in metabolic responses derived from stress system activation. *P-values* determined by two-way ANOVA analysis for each factor of variation, and also the interaction between factors, are showed in Supplementary File 3. No mortality occurred, and no changes in behavior or clinical effects due to anesthetics administration were determined in fish during transport or recovery period.

#### HPI Axis Regulation

**Figure 1:**
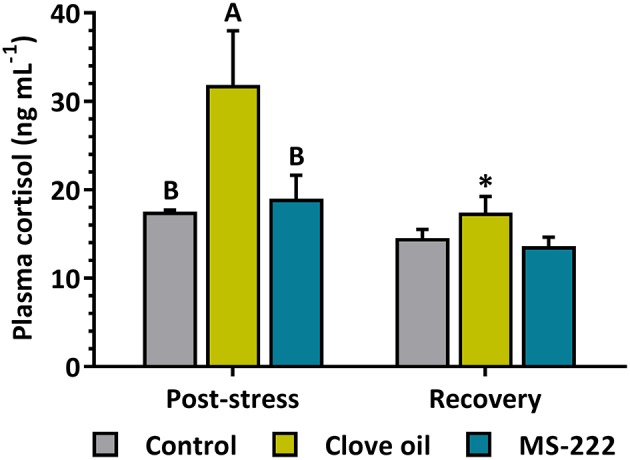
Plasma cortisol (ng mL^−1^) in *S. aurata* juveniles after 6 h of simulated transport (post-stress), under sedation doses of either: (i) control (none); (ii) clove oil (2.5 mg L^−1^); or MS-222 (5 mg L^−1^), and after 18 h of maintenance in clean and quite recovery tanks (recovery). Data are shown as mean ± SEM (*n* = 12). Different capital letters represent statistical differences among sedating conditions after simulated transport; and asterisks represent statistical differences between sampling times for each treatment (two-way ANOVA followed by *post hoc* Tukey test). Statistical significance was accepted at *p* < 0.05.

The simulated transport did not affect HPI axis gene expression in brain or pituitary for both tested anesthetics. Brain *crh, crhbp* and *trh* as well as pituitary *pomc*α*1* and *pomc*α*2* mRNA levels remained constant both after transport and recovery (Table 2). In head kidney, *cyp11b1* mRNA levels were reduced in CO-sedated fish (*p* = 0.0099) after transport, while the decrease observed in MS-222-sedated animals was not significant (*p* = 0.0847). On the contrary, after recovery, *cyp11b1* mRNA levels in CO-sedated fish increased significantly (*p* = 0.0005) respect to control animals (*p* = 0.0154); but in MS-222-sedated fish remained constant (Table 2). A similar response was determined for *star* (Table 2), as shown by a positive linear correlation (*r*^2^ = 0.9507; *p* < 0.0001) with *cyp11b1* mRNA levels in head kidney. No changes were determined in the glucocorticoid receptor (*nr3c1*) after transport; but after recovery, contrary to MS-222-sedated animals, in CO-sedated fish its levels were significantly lower (*p* = 0.0066) in comparison to control animals (*p* = 0.0183) (Table 2). In addition, *S. aurata* juveniles transported with a sedation dose of CO enhanced plasma cortisol levels respect to control group (*p* = 0.0018); but in fish transported with MS-222 no changes were observed (*p* = 0.7291). After recovery, plasma cortisol levels in CO-sedated fish decreased significantly (*p* = 0.0017), and no differences were determined between treatments (Figure 1).

**Table 2:** Gene expression levels (relative units) in brain (*crh, crhbp, trh*), pituitary (*pomc*α*1, pomc*α*2*) and head kidney (*star, cyp11b1, nr3c1*) in *S. aurata* juveniles after 6 h of simulated transport (post-stress), under sedation doses of either: (i) control (none); (ii) clove oil (2.5 mg L^−1^); or MS-222 (5 mg L^−1^), and after 18 h of maintenance in clean and quite recovery tanks (recovery).

	**Post-stress**			**Recovery**		
	**Control**	**Clove oil**	**MS-222**	**Control**	**Clove oil**	**MS-222**
**BRAIN**						
*crh*	1.20 ± 0.10	1.05 ± 0.05	1.08 ± 0.10	1.03 ± 0.05	1.20 ± 0.11	0.99 ± 0.07
*crhbp*	0.81 ± 0.09	0.95 ± 0.01	0.96 ± 0.05	1.09 ± 0.09	1.09 ± 0.07	0.91 ± 0.08
*trh*	1.11 ± 0.06	0.89 ± 0.07	0.90 ± 0.06	1.08 ± 0.11	0.98 ± 0.05	0.94 ± 0.13
**PITUITARY**						
*pomcα1*	0.86 ± 0.13	1.07 ± 0.11	0.94 ± 0.09	1.17 ± 0.13	1.08 ± 0.08	0.95 ± 0.12
*pomcα2*	0.75 ± 0.29	1.22 ± 0.33	0.90 ± 0.17	1.60 ± 0.32	1.22 ± 0.16	1.38 ± 0.33
**HEAD KIDNEY**						
*star*	1.94 ± 0.54A	0.41 ± 0.09B	0.78 ± 0.26B	1.04 ± 0.24b^*^	1.94 ± 0.22a^*^	0.83 ± 0.15b
*cyp11b1*	1.43 ± 0.34A	0.35 ± 0.09B	0.73 ± 0.24AB	0.93 ± 0.18b	1.81 ± 0.36a^*^	0.77 ± 0.18b
*nr3c1*	1.05 ± 0.04	1.01 ± 0.06	0.99 ± 0.05	0.98 ± 0.03a	0.81 ± 0.06b^*^	0.91 ± 0.04ab

*Data are shown as mean ± SEM (n = 12). Different capital letters represent statistical differences among sedating conditions after simulated transport; different lowercase letters represent statistical differences among treatments after recovery; and asterisks represent statistical differences between sampling times for each treatment (two-way ANOVA followed by post hoc Tukey test). Statistical significance was accepted at p < 0.05*.

#### Plasma Parameters

Sedation doses of CO and MS-222 did not alter plasma glycaemia after 6 h of simulated transport. Although plasma levels in non-sedated fish were reduced after the recovery period (*p* = 0.0078), no differences between treatments were determined (Table 3). Plasma lactate levels were not affected by anesthetics addition; but after recovery, lactate levels strongly decreased in all sedation conditions (*p* < 0.0001 for all treatments) (Table 3). *S. aurata* juveniles sedated with CO did not present significant variations in plasma free amino acids after transport; but levels decreased after recovery period (*p* = 0.0172), and in comparison to non-sedated fish (*p* = 0.0009). In MS-222-sedated fish, no changes in plasma amino acids were determined (Table 3). Plasma total proteins and ammonia levels remained without significant variations in all groups after transport and recovery period (Table 3). However, amino acids metabolism was modified by anesthetics addition, showing lower concentrations in both treatments after transport (CO: *p* = 0.00456; MS-222: *p* = 0.0273). The simulated transport also altered intermediary metabolism of lipids. As a result, plasma TAG levels decreased in both sedated groups after transport (CO: *p* = 0.0050; MS-222: *p* = 0.0254) (Table 3). After recovery, plasma TAG raised in both treated groups (CO: *p* = 0.0013; MS-222: *p* < 0.0001), but only in MS-222-sedated animals were higher compared to control fish (*p* = 0.0231) (Table 3).

**Table 3:** Plasma glucose (mg dL^−1^), lactate (mg dL^−1^), triglycerides (TAG, mg dL^−1^), total proteins (mg mL^−1^), amino acids (μmol mL^−1^), ammonia levels (μmol mL^−1^), and osmolality (mOsm kg^−1^) in *S. aurata* juveniles after 6 h of simulated transport (post-stress), under sedation doses of either: (i) control (none); (ii) clove oil (2.5 mg L^−1^); or MS-222 (5 mg L^−1^), and after 18 h of maintenance in clean and quite recovery tanks (recovery).

	**Post-stress**			**Recovery**		
	**Control**	**Clove oil**	**MS-222**	**Control**	**Clove oil**	**MS-222**
Glucose	71.52 ± 5.44	68.8 ± 4.40	63.09 ± 4.36	55.57 ± 3.44^*^	57.88 ± 3.36	55.24 ± 1.34
Lactate	12.92 ± 1.61	10.09 ± 1.02	10.35 ± 1.05	2.38 ± 0.27^*^	3.12 ± 0.69^*^	3.59 ± 0.28^*^
TAG	185.0 ± 9.2A	116.6 ± 12.5B	131.8 ± 10.4B	186.8 ± 19.6b	200.7 ± 21.3ab^*^	236.9 ± 16.9a^*^
Proteins	30.76 ± 0.72	30.29 ± 0.81	29.92 ± 0.72	30.00 ± 0.34	30.76 ± 0.65	31.69 ± 0.48
Amino acids	11.66 ± 0.68	9.94 ± 0.86	11.25 ± 0.26	11.27 ± 1.21a	7.10 ± 0.98b^*^	9.35 ± 0.87ab
Ammonia	13.82 ± 2.15	16.98 ± 2.71	19.55 ± 2.97	13.78 ± 2.45	14.05 ± 2.35	21.02 ± 3.22

*Data are shown as mean ± SEM (n = 12). Different capital letters represent statistical differences among sedating conditions after simulated transport; different lowercase letters represent statistical differences among treatments after recovery; and asterisks represent statistical differences between sampling times for each treatment (two-way ANOVA followed by post hoc Tukey test). Statistical significance was accepted at p < 0.05*.

#### Liver Parameters

In liver, glycogen content determined in CO-sedated fish was not significantly reduced after simulated transport, and remained constant after recovery. In MS-222-sedated fish, glycogen content decreased (*p* = 0.0474), but significantly raised up similar to control levels after recovery (*p* = 0.0058) (Table 4). On the contrary, no significant variations were determined in hepatic free glucose levels (Table 4). After recovery, hepatic amino acids content decreased in non-sedated fish (*p* = 0.0383) to similar levels of CO and MS-222 treatments (Table 4). No changes were determined in liver TAG content after the transport (Table 4), but higher hepatic TAG were determined in MS-222-sedated fish after recovery (*p* = 0.0164) (Table 4).

**Table 4:** Glycogen (mg glc g tissue^−1^), free glucose (mg glc g tissue^−1^), TAG (mg g tissue^−1^), amino acids (μmol g tissue^−1^) and enzymatic activities (GP, PK, LDH-o, FBP, ALT, AST, GLDH, G6PDH, GPDH, HADH; U mg prot^−1^), in liver of *S. aurata* juveniles after 6 h of simulated transport (post-stress), under sedation doses of either: (i) control (none); (ii) clove oil (2.5 mg L^−1^); or MS-222 (5 mg L^−1^), and after 18 h of maintenance in clean and quite recovery tanks (recovery).

	**Post-stress**			**Recovery**		
	**Control**	**Clove oil**	**MS-222**	**Control**	**Clove oil**	**MS-222**
**METABOLITES**						
Glycogen	14.31 ± 1.03A	12.35 ± 1.72AB	10.24 ± 0.72B	13.7 ± 1.62	15.62 ± 1.90	16.06 ± 1.22^*^
Free glucose	6.77 ± 0.26	6.54 ± 0.49	5.88 ± 0.50	7.15 ± 0.54	7.03 ± 0.42	6.42 ± 0.82
TAG	38.98 ± 3.37	40.31 ± 7.02	39.32 ± 2.86	34.12 ± 3.32b	35.62 ± 3.54ab	45.95 ± 3.42a
Amino acids	166.4 ± 8.5A	141.3 ± 7.7AB	137.4 ± 10.8B	139.3 ± 7.7^*^	137.7 ± 7.3	134.6 ± 10.6
**ENZYMES**						
GP	1.66 ± 0.09A	1.00 ± 0.20B	1.55 ± 0.16A	1.46 ± 0.11b	3.58 ± 0.16a^*^	1.89 ± 0.26b
PK	1.71 ± 0.12B	2.52 ± 0.29A	2.32 ± 0.12A	1.84 ± 0.11	1.75 ± 0.14^*^	1.82 ± 0.15^*^
LDH-o	0.15 ± 0.01	0.17 ± 0.01	0.14 ± 0.02	0.14 ± 0.02	0.17 ± 0.02	0.16 ± 0.02
FBP	1.56 ± 0.08	1.58 ± 0.09	1.72 ± 0.09	1.53 ± 0.07b	1.78 ± 0.06a	1.67 ± 0.06ab
ALT	15.00 ± 0.33	14.46 ± 1.12	14.36 ± 0.26	16.63 ± 0.55b^*^	18.39 ± 0.53a^*^	16.44 ± 0.30b^*^
AST	14.07 ± 0.72	14.25 ± 0.51	15.58 ± 0.87	16.20 ± 0.47a^*^	13.20 ± 1.50b	15.42 ± 0.30ab
GLDH	12.37 ± 0.38A	9.56 ± 1.18B	12.51 ± 0.40A	9.30 ± 0.54ab^*^	7.89 ± 0.68b	10.63 ± 0.75a
G6PDH	5.03 ± 0.56A	3.71 ± 0.35B	4.57 ± 0.19AB	3.89 ± 0.13b^*^	4.04 ± 0.28b	5.66 ± 0.18a^*^
GPDH	0.75 ± 0.09	0.87 ± 0.11	0.60 ± 0.04	1.07 ± 0.09^*^	0.92 ± 0.16	0.99 ± 0.14^*^
HADH	3.92 ± 0.14AB	4.33 ± 0.11A	3.69 ± 0.26B	4.26 ± 0.10	4.52 ± 0.28	4.21 ± 0.12

*Different capital letters represent statistical differences among sedating conditions after simulated transport; different lowercase letters represent statistical differences among treatments after recovery; and asterisks represent statistical differences between sampling times for each treatment (two-way ANOVA followed by post hoc Tukey test). Statistical significance was accepted at p < 0.05*.

Liver metabolic enzymes were also modified due to anesthetics addition during transport. GP activity after simulated transport was lower in CO-sedated animals (*p* = 0.0023), but significantly enhanced after recovery (*p* < 0.0001) compared to control animals (*p* < 0.0001). However, no changes were determined for this enzymatic activity in MS-222-sedated fish (Table 4). Moreover, hepatic PK activity similarly enhanced in both sedated groups (CO: *p* = 0.0013; MS-222: *p* = 0.0101); but after recovery period, activities were restored as in control animals (CO: *p* = 0.0021; MS-222: *p* = 0.0304) (Table 4). LDH-o activity remained constant in all groups after the transport and the recovery period (Table 4). Hepatic enzyme activities related to amino acids metabolism were also modified. Thus, although AST activity was similar for all treatments after the stress, in non-sedated fish increased after the recovery period (*p* = 0.0459), being higher compared to CO-sedated animals (*p* = 0.0118). No changes were determined for this enzymatic activity in MS-222-sedated fish (Table 4). In the same way, ALT was not altered by transport in any treatment; but after recovery enhanced in all groups (control: *p* = 0.0445; CO: *p* < 0.0001; MS-222: *p* = 0.0190), being higher in CO-sedated fish (*p* = 0.0375) (Table 4). GLDH activity in CO-sedated animals was also reduced by transport (*p* = 0.0081); but after recovery, activity in non-sedated fish was reduced (*p* = 0.0042), and resulted similar for both groups (*p* = 0.1670). No changes were determined in GLDH activity in MS-222-sedated fish (Table 4). FBP activity remained constant after transport in all treatments; but CO-sedated fish presented higher activity of this enzyme after recovery (*p* = 0.0167) (Table 4). Hepatic enzymes from lipid metabolism were also modified due to transport with CO, as shown by the G6PDH decreased activity (*p* = 0.0063), with no changes in those animals sedated with MS-222. Alternatively, after recovery, G6PDH activity in MS-222-sedated fish increased significantly (*p* = 0.0217); being higher compared to non-sedated animals (*p* = 0.0006), in which a significant decrease of this activity was determined (*p* = 0.0205) (Table 4). On the other hand, any significant variations were determined in HADH or GPDH activities, neither after transport nor after recovery (Table 4).

#### Osmoregulatory Parameters

**Figure 2:**
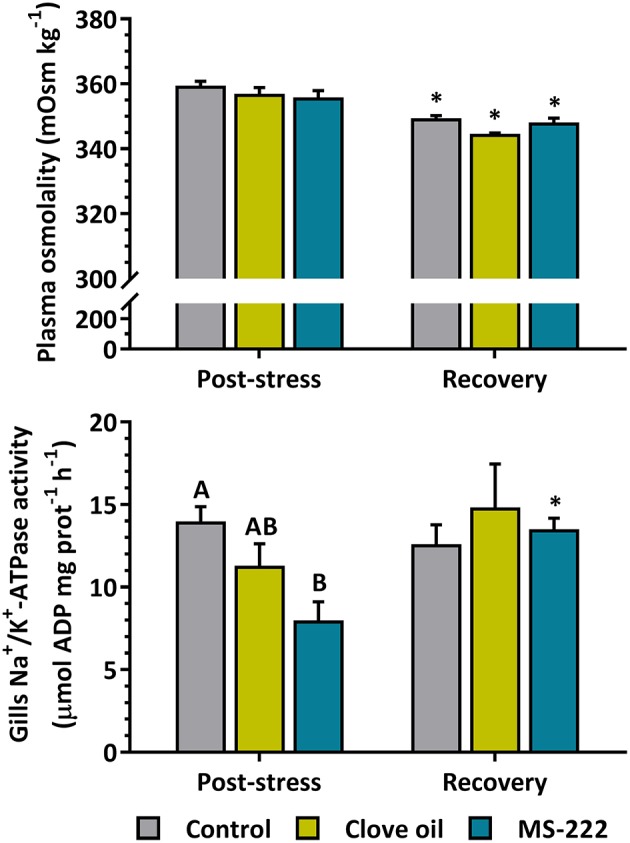
Plasma osmolality (mOsm kg^−1^) and gill NKA activity (μmol ADP mg prot^−1^ h^−1^) in *S. aurata* juveniles after 6 h of simulated transport (post-stress), under sedation doses of either: (i) control (none); (ii) clove oil (2.5 mg L^−1^); or MS-222 (5 mg L^−1^), and after 18 h of maintenance in clean and quite recovery tanks (recovery). Different capital letters represent statistical differences among treatments after recovery; and asterisks represent statistical differences between sampling times for each treatment (two-way ANOVA followed by *post hoc* Tukey test). Statistical significance was accepted at *p* < 0.05.

Plasma osmolality after 6 h of transport was not affected by anesthetics addition; but after recovery decreased in all the treatments (control: *p* = 0.0008; CO: *p* < 0.0001; MS-222: *p* = 0.0079) (Figure 2). On the other hand, gill Na^+^/K^+^-ATPase activity (NKA) after stress was reduced in animals with the sedation dose of MS-222 (*p* = 0.0108), but not in CO-sedated fish (*p* = 0.1973). After the recovery period, MS-222-sedated animals recovered similar values (*p* = 0.0179) as to control and CO groups (Figure 2).

## Discussion

Exposure of gilthead seabream (*Sparus aurata*) juveniles to clove oil (CO) and MS-222 evoked a differential response on the induction to anesthesia progress. In consequence, the use of these agents during a simulated transport process differentially regulated the HPI axis at peripheral level. Our results also showed that the metabolic rearrangement of carbohydrates, amino acids, and lipids depends on the anesthetic employed.

Prior to transport, fish were exposed to different concentrations of CO and MS-222 to characterize timing and doses to induce deep anesthesia, and also to define the sedative concentration for fish transportation. In teleost fish, the suggested maximum time to induce deep anesthesia by immersion should be < 3 min, while the recovery time should take no more than 5 min (Ross and Ross, 2008). The optimal dose of CO determined for *S. aurata* juveniles was 60 mg L^−1^, in accordance to what was described before for this anesthetic on this species (Mylonas et al., 2005). By employing MS-222, *S. aurata* reached the optimal deep anesthesia at 80 mg L^−1^, similar to previous results reported (Vera et al., 2010).

### Effects on HPI Axis

The simulated transport of *S. aurata* juveniles did not induce changes in HPI axis regulation at central level. Neither CO nor MS-222 induced significant changes in expression of the main neuroendocrine factors in the brain (*crh, crhbp*, and *trh*). In the pituitary, a similar lack of response was registered, since no significant changes induced by transport or anesthetics addition were determined in *pomc*α*1* or *pomc*α*2* levels. This suggests that, after 6 h of transport, fish were acclimated to stress conditions and primary responses of HPI-axis were not reflected at mRNA levels (Robertson et al., 1988). In the same way, anesthetic-doses selected seemed to be not enough to evoke a decrease in HPI-axis central factors after 6 h of stress. A similar response was obtained in silver catfish (*Rhamdia quelen*) transported for 6 h with sedation doses of an essential oil of *Myrcia silvatica* (Saccol et al., 2018). However, peripherally changes were found in HPI axis. So, the decreased *star* expression for both sedated groups after transport observed in head kidney, suggests a depression of HPI response; but the *star* expression enhancement for CO-sedated fish after the recovery time points out at a differential response of stress axis depending on the anesthetic, which can be assumed by different effects of the main compounds of each anesthetic in the central nervous system (Priborsky and Velisek, 2018). Interestingly, the high correlation found (*r*^2^ = 0.9507) between *star* and *cyp11b1* in the experiment highlights the readiness of the interrenal cells, in terms of gene expression, for cortisol synthesis after cholesterol intake into the mitochondria (Hagen et al., 2006; Vijayan et al., 2010). It is described in *S. aurata* that changes in hypothalamic and pituitary factors of the HPI, altogether with plasma cortisol increase, occurs within the first 4 h after an acute stress situation (Skrzynska et al., 2018). This may explain the lack of changes in the present study, as fish had undergone a stressful challenge during transport, while recovered their basal levels of these parameters after 6 h. As plasma cortisol increased in the CO-group after transport (6 h after the start of the experiment), coinciding with lower expression of *cyp11b1* in the head kidney, we can postulate that CO may induce differential time responses to stress. After 18 h of recovery, time enough for this species to recover from an acute-stress situation (Skrzynska et al., 2018), fish shown similar cortisol levels in all treatments. However, CO-sedated fish shown that *cyp11b1* expression was stimulated and *nr3c1* decreased. These results could be associated to an additional stress originated by CO, related to increased cortisol levels as other authors reported for this species (Tort et al., 2002). In our experiment, MS-222-sedated fish did not show variations in neither plasma cortisol nor *cyp11b1* levels, but there were modifications in the intermediary metabolism as Molinero and Gonzalez (1995) reported before.

### Effects on Intermediary Metabolism

Metabolic responses derived from the HPI axis activation have been described in other studies related to sedated fish transport (Toni et al., 2013; Zeppenfeld et al., 2014; Vanderzwalmen et al., 2018). In our study, the addition of CO and MS-222 has originated a differential rearrangement of energy sources in *S. aurata* juveniles. Plasma and liver metabolites, as well as hepatic enzyme activities, were modified due to anesthetics after a transport simulation and recovery period. These changes depend not only on the anesthetic employed but also on the metabolic pathways assessed.

#### Carbohydrates Related Metabolism

Plasma glucose and lactate are considered primary stress biomarkers, and increased levels are associated to HPI axis activation in fish (Faught et al., 2016). Glucose and lactate decrease in control group after recovery supports the effectiveness of transport simulation on the stress axis activation with a clearance of these metabolites from the blood for fuel supply in several energy-demanding tissues, but no differences were associated to the use of anesthetics. Nevertheless, described changes in hepatic carbohydrates reinforce the idea of energy expenditure imposed by transport and anesthetics. Thus, glycogen levels in the control group after transport and recovery are comparatively lower than those described in this species (Sangiao-Alvarellos et al., 2005; Skrzynska et al., 2018). These low levels could be associated to the acute stress and starving conditions in this experiment (Sangiao-Alvarellos et al., 2005; Skrzynska et al., 2018), or even to seasonal variations in hepatic energy stores (Vargas-Chacoff et al., 2009). In this study, PK activity enhancement in both sedated groups after transport is clearly associated to an increase in glycolysis, and thus energy production. Additionally, MS-222-sedated fish presented lower hepatic glycogen levels after transport, related to glucose demand increase due to HPI axis response (Vijayan et al., 2010; López-Patiño et al., 2014). It should be highlighted that hepatic free glucose values did not vary significantly due to transport and remained stable after the recovery. This remarks the potential to maintain glucose homeostasis despite the increase in energy requirements. Similarly, CO-sedated fish restored glycogen and free glucose levels after recovery. Even so, GP activity enhancement determined in recovered CO-treated fish points out at a tissue preparation to further energy demand, and the additional stress evoked by CO addition (Tort et al., 2002).

#### Amino Acids Related Metabolism

Proteins and amino acids are important sources of non-carbohydrate substrates for gluconeogenesis, and have been described as hepatic energy fuels in fish under different stress situations (Polakof et al., 2006; Vijayan et al., 2010). In this case, the addition of both anesthetics did not change plasma amino acids values after the stress. In the same way, neither AST and ALT activities (key enzymes in amino acids degradation), nor FBP (involved in gluconeogenic pathways) were modified after transport due to anesthetics addition. Despite this, hepatic amino acids content in both sedated groups was reduced; thus, it is difficult to elucidate the effectiveness of the sedation doses in amino acid catabolism. However, CO addition evoked a differential response in recovered fish, probably related to GLDH activity reduction shown after transport. The decrease in plasma amino acids and liver AST activity in CO-sedated fish, concomitantly to liver ALT and FBP activities increases suggest an enhancement of gluconeogenic pathways during recovery (Polakof et al., 2006). Contrarily, use of MS-222 did not affect amino acid metabolism in recovered fish.

#### Lipids Related Metabolism

Lipid mobilization is also related to HPI activation, mostly due to triglycerides (TAG) allocation as energy substrates for gluconeogenesis pathways in fish (Sheridan, 1988; Faught et al., 2016). In this case, CO and MS-222 addition stimulated TAG consumption in *S. aurata* juveniles, as the reduced plasma levels suggested. For both sedated groups, additional lipid requirement is suggested, but no changes in hepatic TAG content were determined. Therefore, exportation to muscle is suggested as energy substrates for this tissue (Vijayan et al., 1991). Additionally, MS-222-sedated fish presented a compensatory accumulation of TAG after the recovery. That is reflected by the increase in plasma and liver content, and also in liver G6PDH activity, which provides reducing equivalents for fatty acid synthesis (Tocher, 2003).

### Effects on Osmoregulation

Transport processes have been demonstrated to alter osmoregulation in fish by the increase of ion fluxes through the gills, originating a net loss of ions (mainly sodium and chloride), and also accumulation of total ammonia in plasma (Azambuja et al., 2011; Becker et al., 2016). Slight sedation with essential oil of *Aloysia triphylla* in transported *R. quelen* has been reported to reduce these effects (Zeppenfeld et al., 2014). In this case, neither CO nor MS-222 induced changes in plasma osmolality after transport, but *S. aurata* juveniles presented lower levels in all groups after the recovery. This is in accordance to ion loss described in freshwater species as a consequence to stress exposure (Wendelaar Bonga, 2011), but anesthetics addition did not improve this response. Furthermore, the strong reduction in gill Na^+^/K^+^-ATPase (NKA) activity determined in MS-222-sedated fish after the transport could be related to increased plasma ammonia levels, but no significant differences were found. Ammonia cations are expelled mainly through the gills in teleost fish by, among others, ATP-consuming ion transporters such as the Na^+^/H^+^-exchanger (NHE) that can exchange NH4^+^ instead of H^+^ (Quijada-Rodriguez et al., 2017). Altogether, this information suggests that other osmoregulatory mechanisms such as the V-type H^+^-ATPase or the co-transporter Na^+^/K^+^/2Cl^−^ (NKCC) located not only in the gills, but also in other tissues such the intestine (Gregorio et al., 2013; Ruiz-Jarabo et al., 2017), are in co-operation with the branchial NKA to maintain plasma osmolality levels during transport.

### Summary and Future Implications

**Figure 3:**
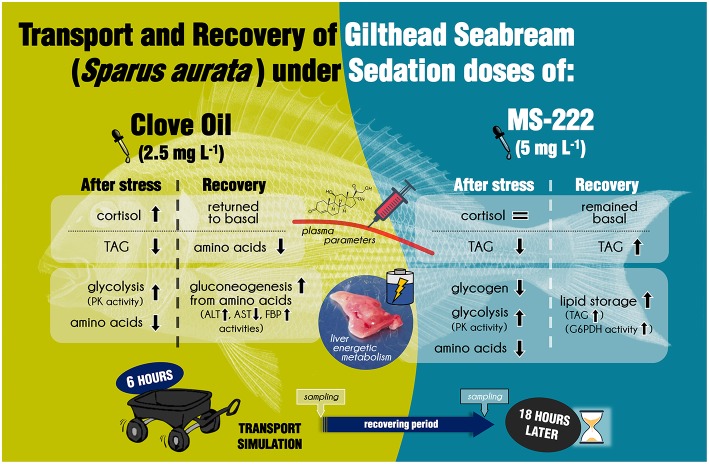
Graphical summary of the main physiological effects determined in *S. aurata* juveniles after simulated transport, and 18 h after its recovery, under sedation doses of either clove oil or MS-222.

Literature is enriched with studies focused on the characterization of anesthesia induction with clove oil and MS-222 in fish. Likewise, many physiological changes related to both anesthetics exposure, have been also described for *S. aurata* and other important aquaculture species (reviewed in Priborsky and Velisek, 2018). In addition, the stress response associated to transport is closely linked to duration of the process and water quality, and also to the anesthetic dose if sedation is implemented (Sampaio and Freire, 2016; Vanderzwalmen et al., 2018). In this way, the simulation performed in this work, with the doses selected of CO (2.5 mg L^−1^) and MS-222 (5 mg L^−1^), has resulted in a useful tool to address the use of these anesthetics in aquaculture practices. To summarize, the decrease of *star* and *cyp11b1* mRNA levels detected in head kidney for both sedated groups could be associated to a stress response inhibition. Nevertheless, the increase in plasma cortisol of CO-sedated fish, and the rebound in the expression of head kidney factors after recovery, shows a negative effect of this anesthetic. In conclusion, regarding HPI regulation, the dose of MS-222 employed could be enough to avoid and additional increase of cortisol levels; but for CO, the sedation dose evokes and additional response of interrenal cells that remained after recovery. This could be related to the faster excretion of MS-222 in comparison to eugenol (main compound of CO) (Priborsky and Velisek, 2018). Additionally, side-effects of anesthetics were described in the intermediary metabolism of *S. aurata* juveniles (summarized in Figure 3). Both anesthetics, in the doses employed herein, increased liver glycolysis and also reduced hepatic amino acids content and plasma TAG, suggesting energy requirements enhancement during transport procedure. Furthermore, liver gluconeogenesis through amino acids catabolism was enhanced in CO-sedated fish, while in MS-222-sedated animals' recovery was associated to lipids consumption. Again, this could be related to the different metabolic dynamics of both chemicals in the organism. To conclude, neither clove oil nor MS-222 improved the stress response in *S. aurata* juveniles during transport with the information derived from this study. Moreover, side-effects on intermediary metabolism were evoked and reflected even 18 h later. Although the use of these agents is not dismissed, further studies should consider other doses of sedation, and analyze HPI modifications throughout the transportation process. Recovery of fish should also be addressed for prolonged periods, in order to evaluate how side-effects alter feed intake or growth rates of reared species.

## Ethics Statement

Fish were kept and handled following the guidelines for experimental procedures in animal research from the Ethics and Animal Welfare Committee of the University of Cadiz, according to the Spanish (RD53/2013) and European Union (2010/63/UE) legislation. The Ethical Committee from the Autonomous Andalusian Government approved the experiments (Junta de Andalucía reference number 28-04-15-241).

## Author Contributions

JM, IR-J, and IJ-C conceived and designed the study. MF-C, TDSO, and IJ-C carried out the experimental procedures. IJ-C, MF-C and IR-J analyzed and interpreted the data. JM-S and GM-R supported molecular biology analysis. IJ-C, IR-J, and JM wrote the original draft. All authors have critically reviewed, edited and approved the final manuscript.

### Conflict of Interest Statement

The authors declare that the research was conducted in the absence of any commercial or financial relationships that could be construed as a potential conflict of interest.

## Abbreviations

MS-222: Tricaine Methanesulfonate
CO: Clove oil
HPI: Hypothalamic-Pituitary-Interrenal axis
CRH: Corticotrophin Releasing Hormone
CRHBP: Corticotrophin Releasing Hormone Binding Protein
TRH: Thyrotropin Releasing Hormone
POMC: Proopiomelanocortin
ACTH: Adenocorticotropic Hormone
StAR: Steroidogenic Acute Regulatory protein
Cyp11b1: 11β-hydroxylase
Nr3c1: glucocorticoid receptor
TAG: Triglycerides
GP: glycogen phosphorylase (EC 2.4.1.1)
PK: pyruvate kinase (EC 2.7.1.40)
GLDH: glutamate dehydrogenase (EC 1.4.1.2)
AST: aspartate aminotransferase (EC 2.6.1.1)
ALT: alanine aminotransferase (EC 2.6.1.2)
G6PDH: glucose-6-phosphate dehydrogenase (EC 1.1.1.49)
HADH: 3-hydroxyacyl-CoA dehydrogenase (EC 1.1.1.35)
GPDH: glycerol-3-phosphate dehydrogenase (EC 1.1.1.8)
FBP, fructose 1: 6-bisphosphatase (EC 3.1.3.11)
LDH-o: lactate dehydrogenase-oxidase (EC 1.1.1.27)
NKA: Na^+^/K^+^-ATPase.
