# Supplementary material for: Transport and Recovery of Gilthead Seabream (Sparus aurata L.) Sedated With Clove Oil and MS-222: Effects on Stress Axis Regulation and Intermediary Metabolism
Source: Front Physiol. 2019 May 31;10:612. doi: 10.3389/fphys.2019.00612 (PMC6555194; doi:10.3389/fphys.2019.00612)
Supplement: Supplementary file 1 [file Data_Sheet_1.pdf]

SUPPLEMENTARY FILES

Supplementary File 1. Sedation, anesthesia and recovery stages defined for *S. aurata* during the exposure to clove oil (CO) and MS-222. Adapted from Ross and Ross (2008).

Induction Stage	Description
Light Sedation	Partial loss of motion, active response to *stimuli*
Deep Sedation	Erratic movements, ventilation decreased, response to *stimuli* reduced
Light Anesthesia	Partial loss of equilibrium, analgesia
Deep Anesthesia	Total loss of equilibrium, no reaction to caudal press, ventilation almost ceased
Recovery	Equilibrium, motion, and perception to *stimuli* completely restored

Supplementary File 2. Specific nucleotide sequences designed as primers for qPCR expression analysis, size of amplified products, concentration of primers used, and efficiencies (*E,* %) and R^2 of curves assessed in *S. aurata*.

Primers	Nucleotide Sequences	Amplicon size (bp)	Concentration (nM)	Tissue	*E* (%)	R^2
$actb_F$	5′-TCTTCCAGCCATCCTTCCTCG-3′	108	200	Brain	100.9	1.000
$actb_R$	5′-TGTTGGCATACAGGTCCTTACGG-3′			Pituitary	94.8	0.998
				Head Kidney	99.4	1.000
$ef1a_F$	5′-AGAGGCTGTCCCTGGTGA-3′	137	200	Brain	102.8	0.999
$ef1a_R$	5′-TGATGACCTGAGCGTTGAAG-3′			Pituitary	102.4	0.998
				Head Kidney	97.7	0.999
crh_F	5′-GGAGCTTAAACGGGCTTGAC-3′	97	200	Brain	91.3	0.999
crh_R	5′-TCAAACATGAAGAACGGCTTAA-3′					
$crhbp_F$	5′-TTTGAAGAGCCACAGATCCA-3′	171	200	Brain	96.1	0.994
$crhbp_R$	5′-TCGAGCTTTATTCAGCGACA-3′					
trh_F	5′-GAAACGCTTTTGGGATAACTCC-3′	131	300	Brain	108.1	0.999
trh_R	5′-CGGCGTGACTCTTGTTTATGTT-3′					
$pomca1_F$	5′-AGCCAGAAGAGAGAGCAGTGAT-3′	120	400	Pituitary	101.6	1.000
$pomca1_R$	5′-ATCGGGTCAGAAAACACTCA-3′					
$pomca2_F$	5′-AGCTCGCCAGTGAGCTGT-3′	81	600	Pituitary	99.4	1.000
$pomca2_R$	5′-CCTCCTGCATCACTTCCTG-3′					
$star_F$	5′-GAGCACAGATGTTGGCTTCA-3′	103	300	Head Kidney	101.1	0.999
$star_R$	5′-GAAACAATCGAGGCACACAA-3′					
$cyp11b1_F$	5′-GCTCTGACGCACACTGAGAA-3′	135	200	Head Kidney	106.5	0.997
$cyp11b1_R$	5′-ATGAGGAAGCCACAATAACAGC-3′					
$nr3c1_F$	5′-TGCTGGCGGAGATCATCACCA-3′	182	200	Head Kidney	93.8	0.999
$nr3c1_R$	5′-GCAGGCCAAGCGAAGGCTTA-3′					

Supplementary File 3. *P-Values* from two-way ANOVA of parameters measured *S. aurata* juveniles. Anesthetics addition during transport (none, clove oil or MS-222), and sampling time (after 6 h of transport or after 18 h of recovery) were considered as factors of variation (n.s., not significant).

Tissue	Parameter	Anesthetic	Time	Anesthetic x Time
Brain	*crh* expression	n.s.	n.s.	n.s.
	crhbp expression	n.s.	n.s.	n.s.
	trh expression	n.s.	n.s.	n.s.
Pituitary	*pomcα1* expression	n.s.	n.s.	n.s.
	pomcα2 expression	n.s.	n.s.	n.s.
Head Kidney	*star* expression	n.s.	n.s.	0.0007
	cyp11b1 expression	n.s.	n.s.	0.0027
	nr3c1 expression	n.s.	0.0058	n.s.
Plasma	Cortisol levels	0.0077	0.0031	n.s.
	Glucose levels	n.s.	0.0006	n.s.
	Lactate levels	n.s.	< 0.0001	n.s.
	TAG levels	n.s.	<0.0001	0.0068
	Proteins levels	n.s.	n.s.	n.s.
	Amino acids levels	0.0041	0.0158	n.s.
	Ammonia levels	n.s.	n.s.	n.s.
	Osmolality	n.s.	< 0.0001	n.s.
Liver	Glycogen levels	n.s.	0.02	n.s.
	Free glucose levels	n.s.	n.s.	n.s.
	TAG levels	n.s.	n.s.	n.s.
	Amino acids levels	n.s.	n.s.	n.s.
	GP activity	< 0.0001	< 0.0001	< 0.0001
	PK activity	n.s.	0.0059	0.0237
	LDH-o activity	n.s.	n.s.	n.s.
	FBP activity	n.s.	n.s.	n.s.
	ALT activity	n.s.	< 0.0001	n.s.
	AST activity	n.s.	n.s.	n.s.
	GLDH activity	0.0008	0.0005	n.s.
	G6PDH activity	0.0023	n.s.	0.0053
	GPDH activity	n.s.	0.0084	n.s.
	HADH activity	0.0420	0.0251	n.s.
Gills	NKA activity	n.s.	0.0438	n.s.
